# Foam cells promote atherosclerosis progression by releasing CXCL12

**DOI:** 10.1042/BSR20193267

**Published:** 2020-01-17

**Authors:** Lingxing Li, Zhenlan Du, Bing Rong, Dapeng Zhao, Aiping Wang, Yuzhen Xu, Huanyi Zhang, Xue Bai, Jingquan Zhong

**Affiliations:** 1 The Key Laboratory of Cardiovascular Remodeling and Function Research, Chinese Ministry of Education and Chinese Ministry of Health, The State and Shandong Province Joint Key Laboratory of Translational Cardiovascular Medicine, Qilu Hospital of Shandong University, Jinan, China; 2 Department of Cardiovascular Medicine, Tai’an City Central Hospital, Taian, China; 3 Department of Neurology, Tai’an City Central Hospital, Taian, China

**Keywords:** Atherosclerosis, CXCL12, foam cell, macrophage

## Abstract

**Background:** Atherosclerosis (AS) is a chronic inflammatory disease that contributes to multiple cardiovascular diseases (CVDs), and foam cell formation plays important roles in the progression of AS. There is an urgent need to identify new molecular targets for treating AS, and thereby improve the quality of life and reduce the financial burden of individuals with CVD.

**Methods:** An *in vitro* model of AS was generated by treating THP-1 cells and human aortic vascular smooth muscle cells (HA-VSMCs) with oxidized low-density lipoproteins (ox-LDLs). HA-VSMC proliferation and foam cell formation were detected by the MTT assay and Oil Red O staining. C–X–C motif chemokine 12 (CXCL12) expression was suppressed by siRNA. An AS rat model was established by feeding rats a high-fat diet and vitamin D2 for 3 weeks. Histopathology examinations were conducted by Hematoxylin and Eosin (H&E) staining and the levels ionized calcium-binding adapter molecule 1 (IBA1) and α smooth muscle actin (α-SMA) expression were determined by ELISA assays and immunohistochemistry.

**Results:** An *in vitro* model of AS was established with THP-1 cells. CXCL12 expression in the model THP-1 cells was significantly increased when compared with its expression in control cells. Suppression of CXCL12 expression reduced the progression of AS in the cell model. Moreover, CXCL12 promoted AS in the *in vivo* rat model.

**Conclusion:** Our results suggest that CXCL12 plays an important role in promoting the progression of AS. Furthermore, inhibition of CXCL12 might suppress the development of AS by inhibiting HA-VSMC proliferation and their transformation to foam cells.

## Introduction

Atherosclerosis (AS) is a multi-step process that causes cardiovascular diseases (CVDs) by obstructing blood flow to the heart, brain or lower extremities via luminal stenosis or thrombosis [6]. Dysfunction of endothelial cells is the initial event in AS, and is followed by the release of low-density lipoproteins (LDLs), invasion of macrophages and vascular smooth muscle cells (VSMCs), foam cell formation, and extracellular matrix remodeling, all of which complete the AS process [31]. In the present study, we examined the molecular mechanisms involved in atherosclerotic plaque formation, with the goal of identifying new methods for treating AS.

The phenotypic switching of VSMCs has long been considered of fundamental importance to AS, which expresses macrophage markers and properties [32]. Suppression of VSMC phenotypic switching might be useful for treating cases of advanced AS [1]. The turnover rate of VSMCs in normal vessel walls is low, and the VSMC proliferation index is difficult to measure [37]; however, it is known that cell proliferation becomes increased during the early stage of atherogenesis [5]. On the other hand, the proliferation of VSMCs is mainly reparative in atherogenesis; therefore, an increase in VSMC proliferation during the early stage of atherogenesis might be beneficial. However, no study has shown that direct intervention can avoid the phenotypic switching of VSMCs [3].

C–X–C motif chemokine 12 (CXCL12), also known as SDF-1 (stromal cell-derived factor-1), is a chemokine protein that binds to two receptor proteins (CXC chemokine receptor 4 [CXCR4] and CXCR7) to regulate various biological processes [18]. Previous studies showed that CXCL12 is highly expressed in the pathological conditions associated with AS, including hyperlipidemia, pro-inflammatory responses, VSMC proliferation/migration and insulin resistance [8,15,33,34,39]. In addition, a high expression of CXCL12 and its receptors in carotid atherosclerotic plaque is closely related to the progression of AS [16,24,27]. However, the exact role of CXCL12 in AS is not fully understood.

In the present study, we used phorbol myristate acetate (PMA) to induce THP-1 cells to form macrophages and used oxidized low-density lipoproteins (ox-LDLs) to induce macrophages to form foam cells as a cellular model of AS. We next examined CXCL12 expression in the cellular model of AS and co-cultured ox-LDL-treated THP-1 cells with HA-VSMCs to determine the cell proliferation and foam cell formation capabilities of HA-VSMCs. Additionally, we used siRNA to suppress CXCL12 expression as a means of testing the effects of CXCL12 in the AS cell model. Moreover, CXCL12 expression was determined in *in vivo* using a rat AS model.

## Materials and methods

### Cell culture

Human TPH1 monocytic cells and human aorta VSMCs were cultured in RPMI 1640 medium supplemented with 10% heat-inactivated FBS (HyClone, Logan, UT, U.S.A.), 1% penicillin and 1% streptomycin. The Key Laboratory of Cardiovascular Remodeling and Function Research, Chinese Ministry of Education and Chinese Ministry of Health, The State and Shandong Province Joint Key Laboratory of Translational Cardiovascular Medicine, Qilu Hospital of Shandong University, at 37°C in a 5% CO_2_ incubator. The cells were then treated with 100 ng/ml of PMA (Sigma–Aldrich, St. Louis, MO, U.S.A.) for 48 h to induce their differentiation to macrophages.

### Immunofluorescence

HA-VSMCs (2 × 10^5^) were seeded on to coverslips in a 12-well plate and cultured overnight. After fixation with paraformaldehyde (4%), the cells were permeabilized with 0.1% Triton X-100 and then blocked with 2% BSA. The cells were then incubated overnight at 4°C with a primary body against α smooth muscle actin (α-SMA) (A5228, Sigma, 1:200), followed by incubation with an Alexa Fluor 488-labeled secondary antibody (4408, Cell Signaling Technology, Danvers, MA, U.S.A., 1:500) for 1 h at room temperature. The cell nuclei were visualized by staining with DAPI. Finally, images of the stained cells were collected with a laser scanning confocal microscope (ZEISS LSM 710, Carl Zeiss, AG, Germany).

### Co-culture system

HA-VSMCs were seeded into the lower chamber of a Transwell plate (3422, Corning, Corning, NY, U.S.A.), and THP-1 cells were seeded on to the upper chamber. The THP-1 cells were then treated with ox-LDLs. Next, the HA-VSMCs and THP-1 cells were cultured for 24 or 48 h.

### Cell proliferation assay

HA-VSMCs (1 × 10^4^) were seeded on to the lower chamber of a Transwell plate (3422, Corning), and THP-1 cells (1 × 10^4^) were seeded on to the upper chamber. Next, the THP-1 cells were treated with ox-LDLs. After 24 or 48 h, the upper chamber was removed, and 100 µl of MTT (V13154, Thermo Fisher, Waltham, MA, U.S.A.) was added to the HA-VSMCs, which were then cultured for another 2 h. Finally, 500 µl of DMSO was added and the absorbance at 490 nm was determined with a microplate reader (iMark, Bio-Rad, Hercules, CA, U.S.A.). Each experiment was repeated three times.

### ELISA for CXCL12

After 48 h of incubation, the cell culture supernatant was collected (for the co-culture system, THP-1 cells were co-cultured with HA-VSMCs; after 48 h, the THP-1 cells were removed and the culture medium in the bottom chamber was collected), and the ELISA was performed with an ELISA kit (DSA00, R&D Systems, Minneapolis, MN, U.S.A.).

### Oil Red O Staining

Cells (3 × 10^5^) were cultured overnight on slides and subsequently treated with ox-LDLs (50 mg/l) for the indicated time. After fixation with 4% paraformaldehyde, the cells were stained with 0.3% Oil Red O for 20 min, and images were collected with a Zeiss microscope (Imager A2, Carl Zeiss Microscopy, Germany).

### Western blotting

Samples of total protein (30 μg each) extracted from THP-1 cells or VSMCs were separated by 10% SDS/PAGE. Next, the protein bands were transferred on to PVDF membranes (Millipore, Burlington, MA, U.S.A.), which were subsequently blocked with 5% non-fat milk. The membranes were then incubated with a primary antibody against CXCL12 (3740, Cell Signaling Technology, 1:1000) or GAPDH (97166, Cell Signaling Technology, 1:2000) at 4°C overnight. The next morning, the membranes were washed three times with 1× TBST, and then incubated with HRP–conjugated goat anti-mouse (7076, Cell Signaling Technology, 1:5000) or anti-rabbit (7074, Cell Signaling Technology, 1:5000) IgG at room temperature for 1 h. The immunostained protein bands with detected with an ECL substrate (Thermo Scientific).

### RNA isolation and quantitative real-time PCR

TRIzol was used to extract the total RNA from cells. Next, samples of total RNA were reverse transcribed to cDNA with an M-MLV reverse transcriptase kit (TaKaRa Co., Dalian, China). The relative levels of GAPDH and CXCL12 expression were determined by real-time PCR performed with a SYBR Green qPCR kit (TaKaRa Co., Dalian, China). The primer sequences used were as follows:

CXCL12 F-5′- TACAGATGCCCATGCCGATT-3′
CXCL12 R-5′-CTGAAGGGCACAGTTTGGAG-3′
GAPDH F-5′ -TGTTCGTCATGGGTGTGAAC-3′
GAPDH R-5′ -ATGGCATGGACTGTGGTCAT-3′.

### SiRNA transfection

Cells were plated into a six-well plate and cultured to 30–50% confluence; after which they were transfected with siRNA by using Lipofectamine 2000 (Invitrogen, Carlsbad, CA, U.S.A.) according to the manufacturer’s instructions.

The CXCL12 siRNA sequences were: F: 5 -AUGGCUUUCGAAGAAUCGGCAUGGG-3′, R: 5′-CCCAUGCCGAUUCUUCGAAAGCCAU-3′.

### AS rat model

Male Sprague–Dawley rats (12 weeks old) were purchased from the Animal Center of Shandong University. All animal experiments were conducted at the Experimental Animal Center of Shandong University, and all experimental protocols were approved by the Animal Care and Use Committee of Qilu Hospital of Shandong University. The SD rats were randomly assigned to a control group (*n*=5 rats) and an AS (model, *n*=5 rats). AS was established by feeding rats a high-fat diet plus vitamin D_2_ (3 × 10^5^ U/kg) for 3 weeks as previously described [40].

### Hematoxylin and Eosin staining

After 2 weeks of dietary treatment, the rats were maintained in a fasted state for 8 h prior to being anesthetized with sodium pentobarbital (50 mg/kg weight). The main pulmonary arteries of each rat were separated and fixed. Next, samples of pulmonary artery tissue were embedded in paraffin, sectioned, mounted on to slides, and stained with Hematoxylin and Eosin. Images of the stained tissues were captured with a light microscope.

### Immunohistochemistry

Sections of paraffin-embedded arteries were deparaffinized and then rehydrated. The sections were then blocked with 2% normal goat serum and incubated with IBA (ab5076, Abcam, Cambridge, U.K,. 1:100) or α-SMA (A5228, Sigma, 1:200) antibodies at 4°C, overnight. The next morning, the tissues were incubated for 1 h at room temperature with an HRP–conjugated secondary antibody. Hematoxylin was used for counterstaining.

### Statistical analysis

All data were analyzed using GraphPad Prism 7.0 software, and results were shown as the mean ± SD. Differences between groups were evaluated using Student’s *t* test, and a *P*-value <0.05 was considered to be statistically significant.

## Results

### Establishment and identification of an AS cell model

**Figure 1:**
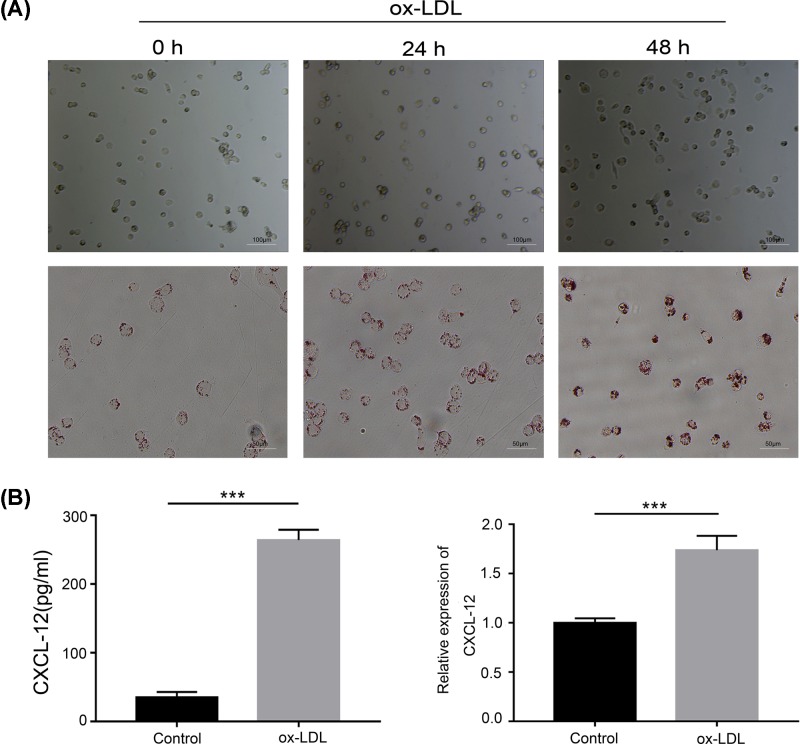
Establishment and identification of an AS cell model (**A**) THP-1 cells were pre-treated with PMA (100 ng) for 48 h to induce their transformation to macrophages. Ox-LDLs were incubated with PMA-treated cells for 0, 24, and 48 h. Cell morphology was detected by light microscopy (upper panel). The cells were fixed and then stained with Oil Red O (lower panel), and the respective images are shown (×200). (**B**) The medium from cultured THP-1 cells was collected and tested for CXCL12 by ELISA (left panel). The cultured cells were collected and RNA was extracted; after which, CXCL12 expression was detected by real-time PCR (right panel). ***, *P*<0.001, control *vs.* ox-LDL.

Foam cells play important roles at all stages of atherosclerotic lesion development [13]. The THP-1 cell is a monocyte model; therefore, we used PMA to induce THP-1 cells to transform into macrophages. Next, we used ox-LDLs to induce macrophages to transform into foam cells as a cell model of AS [4,12]. As shown in Figure 1A, ox-LDLs induced macrophages to form foam cells after 24 h of treatment, as detected by Oil Red O staining. Previous studies found that CXCL12 and its receptors are closely associated with the progression of aAS[16]. We further detected the expression of CXCL12 in our cell model of AS by real-time PCR and ELISA. Our results showed that CXCL12 expression was significantly increased in ox-LDL-treated cells (Figure 1B), suggesting that CXCL12 was induced in the AS cell model.

### Ox-LDLs promoted foam cell formation in HA-VSMCs

**Figure 2:**
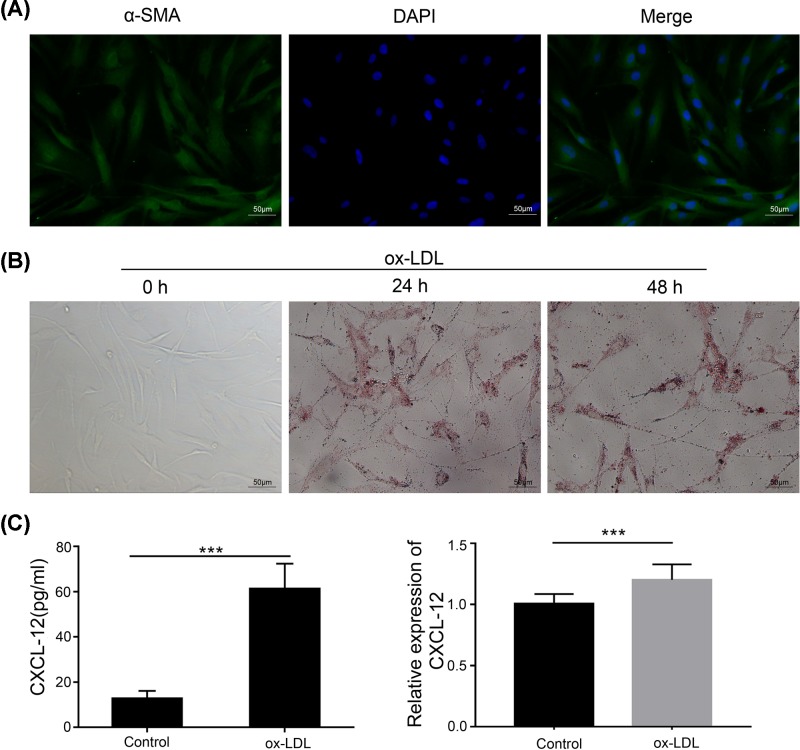
Ox-LDL promoted foam cell formation by HA-VSMCs (**A**) HA-VSMCs were cultured in a 12-well plate. After fixation, the cells were stained with α-SMA antibody, and the respective images are shown (×200). (**B**) HA-VSMCs were treated with ox-LDLs for the indicated times, and foam cell formation was determined by Oil Red O staining. The respective images are shown (×200). (**C**) The medium from cultured HA-VSMCs was collected and tested for CXCL12 by ELISA (left panel). The cultured cells were collected and their total RNA was extracted; after which, CXCL12 expression was detected by real-time PCR (right panel). ***, *P*<0.001, control *vs.* ox-LDL.

The differentiation, migration, and proliferation of VSMCs are involved in the progression of atherosclerotic plaque. Therefore, we treated VSMCs with ox-LDLs to examine foam cell formation by human aortic VSMCs (HA-VSMCs). Immunofluorescence assays showed that most of the cultured HA-VSMCs stained positive for α-SMA, a specific marker for smooth muscle cells, and the cells were spindle-shaped (Figure 2A). Previous studies showed that foam cell formation and monocyte recruitment are essential processes in the pathogenesis of AS [7,38]. We thus used ox-LDLs to induce foam cell formation. Foam cell formation was evaluated by Oil Red O staining, and the results showed that treatment with ox-LDLs had induced accumulations of lipid droplets (Figure 2B). We further detected CXCL12 expression in HA-VSMCs by ELISA and real-time PCR, and found that CXCL12 was significantly increased in ox-LDL-treated HA-VSMCs (Figure 2C).

### Ox-LDL-treated THP-1 cells promoted HA-VSMC proliferation and foam cell formation

**Figure 3:**
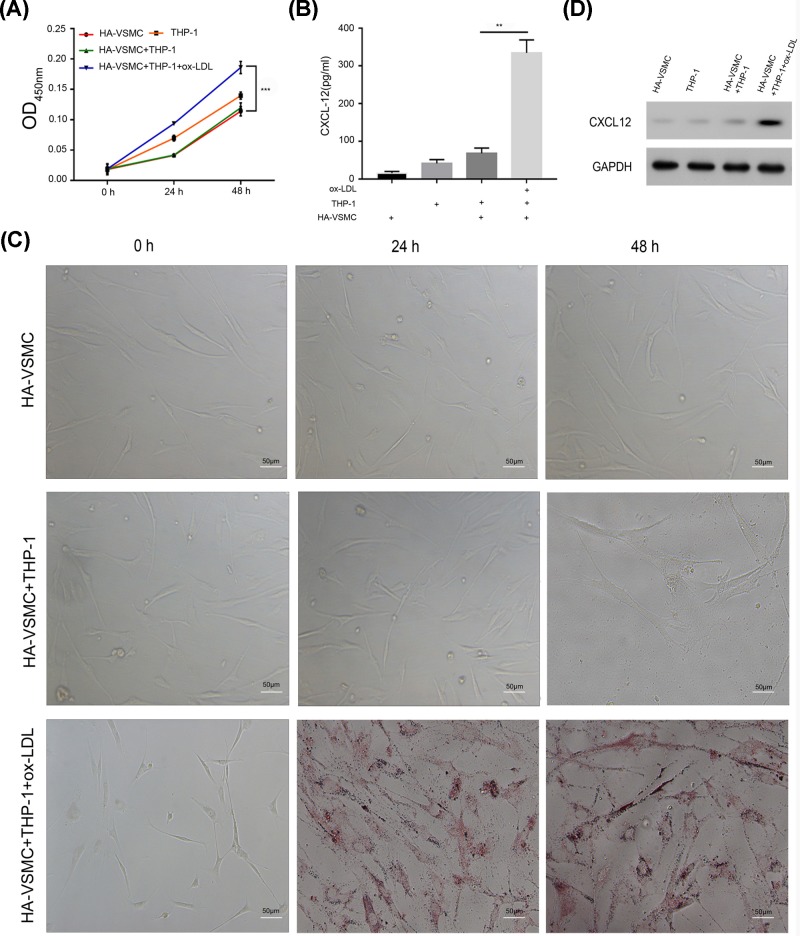
Ox-LDL-treated THP-1 cells promoted HA-VSMC proliferation and foam cell formation (**A**) HA-VSMCs, THP-1 cells, co-cultured HA-VSMCs (bottom chamber) with THP-1 cells (upper chamber), and co-cultured HA-VSMCs (bottom chamber) with ox-LDL-treated THP-1 cells (upper chamber) were seeded into Transwell plates and incubated for 24 or 48 h. The upper chamber was then removed and cell proliferation was detected with the MTT assay. (**B**) Cells were treated as in (A); after 48 h, the upper chamber was removed and the HA-VSMCs were fixed and stained with Oil Red O. The respective images are shown (×200). (**C**) Cells were treated as in (A); after 48 h, the upper chamber was removed, the culture medium in the bottom chamber was collected, and CXCL12 expression was examined by ELISA. (**D**) Cells were collected and lysed with lysis buffer, and Western blotting was performed. **, *P<*0.05, HA-VSMC+THP-1 *vs.* HA-VSMC+THP-1+ox-LDL; ***, *P*<0.001, HA-VSMCs+THP-1 *vs.* HA-VSMCs+THP-1+ox-LDLs.

To test the effects of ox-LDL-treated THP-1 cells on the ability of HA-VSMCs to proliferate and form foam cells, we co-cultured HA-VSMCs with THP-1 cells that had been treated with ox-LDLs. Our results showed that co-culture with ox-LDL-treated THP-1 cells significantly increased the proliferation of HA-VSMC when compared with the proliferation non-co-cultured HA-VSMCs (Figure 3A). Additionally, foam cell formation also was increased among the ox-LDL-treated THP-1 co-cultured VSMCs (Figure 3B). Moreover, we detected CXCL12 expression by ELISA and Western blotting and found that ox-LDL-treated THP-1 significantly increased CXCL12 expression in VSMCs when compared with CXCL12 expression in THP-1 co-cultured HA-VSMCs (Figure 3C,D). These results suggest that CXCL12 released by THP-1 cells may induce the proliferation of HA-VSMC and their transformation to foam cells.

### CXCL12 regulated ox-LDL-treated THP-1 cell-induced HA-VSMC proliferation and foam cell formation

**Figure 4:**
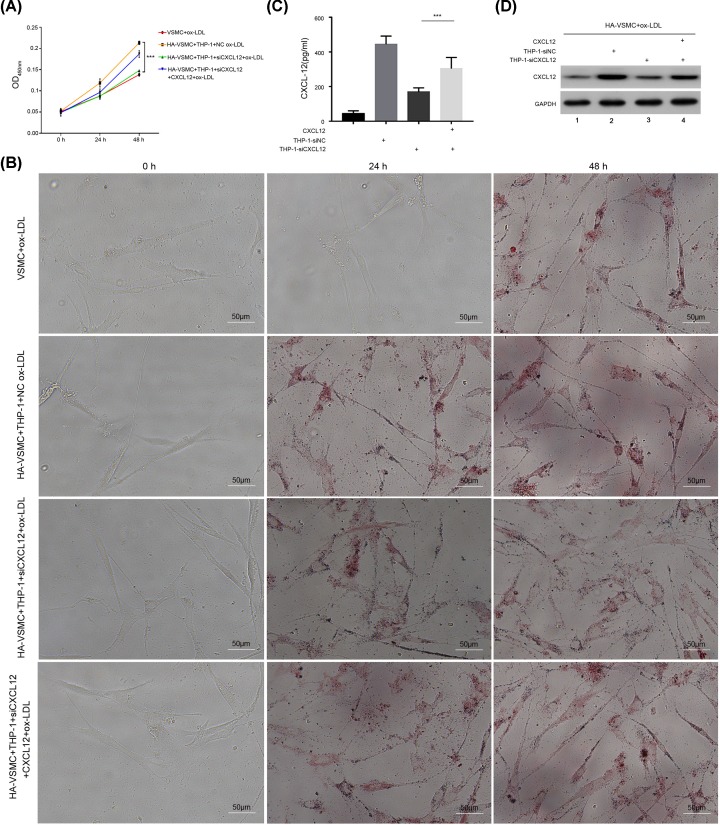
CXCL12 regulated ox-LDL-treated THP-1 cell-induced HA-VSMC proliferation and foam cell formation (**A**) HA-VSMCs treated with ox-LDLs, co-cultured HA-VSMCs (bottom chamber) with ox-LDL-treated THP-1 cells (upper chamber), co-cultured HA-VSMCs (bottom chamber) with CXCL12 siRNA transfected THP-1 cells and treated with ox-LDL (upper chamber), and co-cultured HA-VSMCs (bottom chamber) with CXCL12 siRNA transfected THP-1 cells and treated with ox-LDL and CXCL12 (upper chamber), were seeded into Transwell plates and incubated for the indicated times. The upper chamber was then removed, and cell proliferation was detected with the MTT assay. (**B**) Cells were treated as in (A); after 48 h, the upper chamber was removed, and the HA-VSMCs were fixed and stained with Oil Red O. The respective images are shown (×200). (**C**) Cells were treated as in (A); after 48 h, the upper chamber was removed, the culture medium in the bottom chamber was collected, and CXCL12 was detected by ELISA. (**D**) Cells were treated as in (A); after 48 h, the upper chamber was removed, the HA-VSMCs were collected and lysed with lysis buffer, and Western blotting was performed. ***, *P*<0.001, HA-VSMCs+THP-1-NC siRNA+ox-LDL *vs.* HA-VSMCs+THP-1-CXCL12 siRNA+ox-LDL, HA-VSMCs+THP-1-CXCL12 siRNA+ox-LDL *vs.* HA-VSMCs+THP-1-CXCL12 siRNA+ox-LDL+CXCL12. 1, HA-VSMCs were treated with ox-LDL. 2, THP-1 cells were transfected with NC siRNA, treated with ox-LDLs, and co-cultured with HA-VSMCs. 3, THP-1 cells were transfected with CXCL12 siRNA, treated with ox-LDLs, and co-cultured with HA-VSMCs. 4, THP-1 cells were transfected with CXCL12 siRNA; next, CXCL12 and ox-LDLs were added to the cells, which were then co-cultured with HA-VSMCs.

To estimate the effects of CXCL12 on HA-VSMC proliferation and foam cell formation, we used siRNA to decrease CXCL12 expression in THP-1 cells, and then detected the proliferation of HA-VSMCs with the MTT assay. As shown in Figure 4A, the proliferation of HA-VSMCs in the CXCL12-depleted AS cell model group was significantly attenuated when compared with proliferation in the control siRNA transfected group. Conversely, addition of CXCL12 to CXCL12 siRNA transfected THP-1 cells reversed the proliferative ability of HA-VSMCs (Figure 4A). Moreover, we measured foam cell formation by Oil Red O staining, and found that foam cell formation was also reduced by transfection with CXCL12 siRNA (Figure 4B). Furthermore, CXCL12 expression was detected by ELISA and Western blotting (Figure 4C,D). Our results suggest that CXCL12 regulated the ox-LDL induced proliferation and foam cell formation of HA-VSMCs.

### CXCL12 was increased in the AS rat model

**Figure 5:**
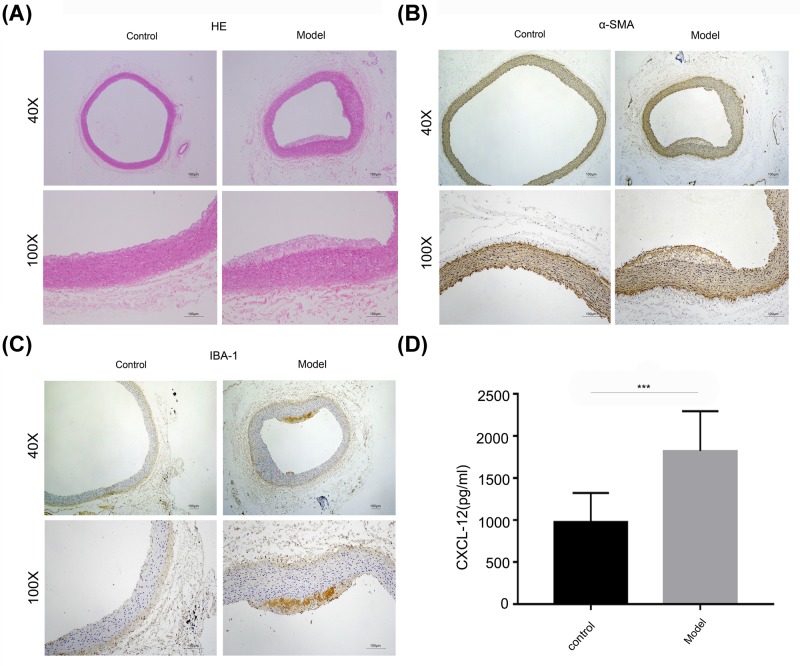
CXCL12 levels were increased in the AS rat model (**A**) The main pulmonary artery regions were collected and stained with H&E. (**B,C**) IBA1 and α-SMA expression were determined by IHC. (**D**) Peripheral blood was collected and tested for CXCL12 by ELISA. ***, *P*<0.001, control *vs.* model.

To evaluate the expression of CXCL12 *in vivo*, we established a rat model of AS as demonstrated by Hematoxylin and Eosin (H&E) staining. The atherosclerotic rats had significantly more extensive lesions in their main pulmonary artery regions when compared with rats in the control group (Figure 5A). The levels of α-SMA and ionized calcium-binding adapter molecule 1 (IBA1) expression were tested by IHC and showed that IBA1 was significantly induced in the AS rat model (Figure 5B,C). Next, samples of peripheral blood were collected and tested for CXCL12 expression by ELISA. The results showed that CXCL12 expression was significantly increased in the AS model rats (Figure 5D). Our data indicate that CXCL12 expression was enhanced in both the *in vivo* AS rat model and the *in vitro* cell model, suggesting that CXCL12 plays essential roles in AS progression.

## Discussion

In the present study, we used HA-VSMCs and THP-1 cells to establish an *in vitro* AS model, and found that CXCL12 expression was significantly increased in the model when compared with the control. Additionally, suppression of CXCL12 reduced the progression of AS in the model cells. Moreover, CXCL12 also enhanced AS progression in a rat model. These results suggest that CXCL12 plays an important role in the progression of AS.

AS has long been known as a progressive chronic inflammatory disease of the arteries [25]. The subendothelial deposition of oxidized LDLs (ox-LDLs), the formation of foamy macrophages, and the proliferation/migration of VSMCs are central pathophysiologic steps in the formation of atherosclerotic plaque [20]. The excessive uptake of ox-LDLs by monocyte-derived macrophages and a decrease in cholesterol outflow are factors that accelerate atherosclerotic plaque formation, and also key processes that determine the size of atherosclerotic plaque [28].

THP-1 cells are the most popular cell line used to explore the functions of monocytes and macrophages in the cardiovascular system [30]. A previous study showed that THP-1 cells have a morphology and differentiation capability similar to those of primary monocytes and macrophages [2]. PMA treatment of THP-1 cells was shown to reduce cell proliferation and induce phagocytosis, which leads to a mature macrophage phenotype [14]. Here, we treated PMA stimulated THP-1 cells with ox-LDLs to establish an *in vitro* cell model of AS, and found that ox-LDLs significantly induced foam cell formation in both THP-1 cells and HA-VSMCs.

The interaction between monocytes and VSMCs promotes the maintenance of subendothelial monocytes and macrophages in AS [28], and may play an essential role in vascular calcification. The Transwell system was used for co-culturing of VSMCs and THP-1 cells to study this process [9]. VSMCs were cultured in the bottom Transwell chamber and THP-1 cells were cultured in the upper chamber to determine the effects of THP-1 cells on the foam cell formation and proliferation of VSMCs. These two cell lines shared the same culture medium. These Transwell co-culture studies showed that ox-LDL-treated THP-1 cells promoted HA-VSMC proliferation and foam cell formation.

The proliferation, migration, and differentiation of VSMCs is involved in atherosclerotic plaque progression, and dysfunctional VSMCs accelerate AS progression [22,26]. When stimulated by a dysfunctional endothelium and inflammatory factors, VSMCs differentiate and proliferate, and form a fibrous cap [17]. Here, we provide evidence that ox-LDLs induced the expression of CXCL12 in PMA-stimulated THP-1 cells and HA-VSMCs. Suppression of CXCL12 in THP-1 cells reduced the proliferation of ox-LDL-treated THP-1 co-cultured VSMCs, and also the transformation of those VSMCs to foam cells, suggesting that CXCL12 released by macrophages contributes to the proliferation of VSMCs.

CXCL12 is an important survival factor for many cell types [10,11,21]. CXCL12 (also known as SDF-1) regulates various cellular activities by binding to CXCR4 or CXCR7 [18]. A previous study showed that human recombinant protein SDF-1 significantly increased the proliferation rate of VSMCs, while an anti-CXCR4 monoclonal antibody blocked the proliferation of VSMCs [23]. Additionally, SDF-1α expression was found to be enhanced in the tunica medias of thoracic aortas obtained from streptozotocin-induced hyperglycemic SD rats, and an SDF-1α neutralizing antibody weakened the high glucose-potentiated proliferation and chemotaxis of VSMCs [19]. Moreover, a study in mice showed that a local femoral artery injury induced local vascular SDF-1 expression, and a disruption of SDF-1/CXCR4 signaling suppressed the proliferative response during vascular remodeling [29]. Platelet-derived CXCL12 is an important chemokine, and CXCR4 and CXCR7 are involved in regulating the functions, survival, and differentiation of monocytes [11]. Previous studies showed that CXCL12 plays a role in AS progression and found elevated serum CXCL12 levels contributed to an increased risk for CVDs [35,36]. Our results are consistent with previous studies, as they showed that CXCL12 expression was significantly increased in an *in vitro* co-cultured cell model and an *in vivo* AS model. Inhibition of CXCL12 release from macrophages reduced the proliferation of VSMCs, suggesting that CXCL12 released from THP-1 cells had stimulated the proliferation of VSMCs, leading to atherosclerotic plaque progression.

CXCL12 has served as a diagnostic and prognostic marker of AS-associated CVD [18]. Because CXCL12 displays pro-atherogenic properties, reducing its circulating levels or inhibiting its production might be a novel strategy for treating AS-associated diseases. However, no specific CXCL12 inhibitor or antibody has been found to prevent the progression of AS. In a subsequent study, we will examine whether inhibition of CXCL12 by a small molecular inhibitor or specific antibody might alleviate AS.

**Figure 6:**
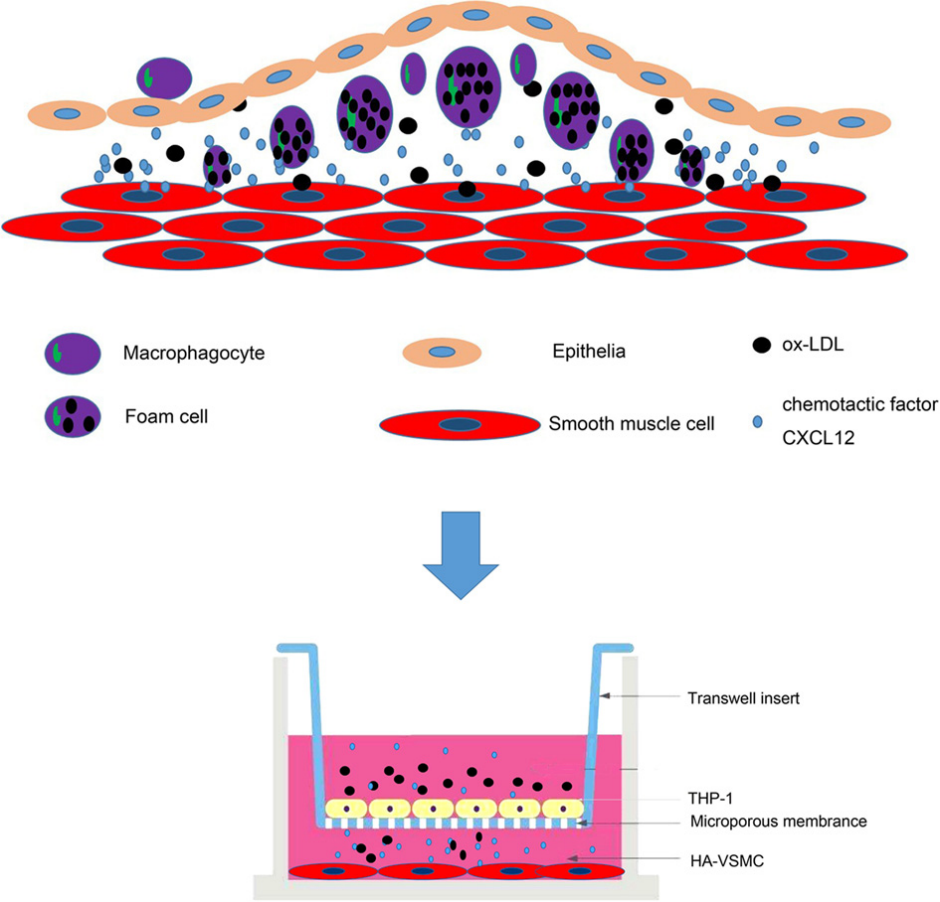
The role of CXCL12 in AS progression AS initiates the infiltration of ox-LDLs into the subendothelial space of arteries, which leads to stimulation of inflammatory infiltration and adhesion between endothelial cells and inflammatory monocytes. Ox-LDLs induced the release of CXCL12 from macrophages and further promoted HA-VSMC proliferation and foam cell formation. Our results demonstrated that CXCL12 expression was significantly increased in the *in vitro* and *in vivo* AS models. Moreover, inhibition of CXCL12 reduced progression in the AS cell model.

In conclusion, our results showed that ox-LDLs induced the release of CXCL12 from macrophages that were previously induced by PMA, and further promoted the proliferation of HA-VSMCs and their transformation to foam cells; these changes caused the formation of atherosclerotic plaque (Figure 6). Our data showed that CXCL12 expression was significantly increased in both an *in vitro* co-cultured cell model and an *in vivo* AS model. Moreover, inhibition of CXCL12 reduced the proliferation of VSMCs. Because CXCL12 promotes AS progression, it may also have a role in treating AS.

## Abbreviations

AS: atherosclerosis
CVD: cardiovascular disease
CXCL12: C–X–C motif chemokine 12
CXCR4/7: CXC chemokine receptor 4/7
HA-VSMC: human aortic vascular smooth muscle cell
IBA1: ionized calcium-binding adapter molecule 1
ox-LDL: oxidized low-density lipoprotein
PMA: phorbol myristate acetate
SDF-1: stromal cell-derived factor-1
VSMC: vascular smooth muscle cell
α-SMA: α smooth muscle actin

## References

[1] Ackers-Johnson M., Talasila A., Sage A.P., Long X., Bot I., Morrell N.W. et al. (2015) Myocardin regulates vascular smooth muscle cell inflammatory activation and disease. Arterioscler. Thromb. Vasc. Biol. 35, 817–828 10.1161/ATVBAHA.114.305218 25614278 PMC4390125

[2] Aldo P.B., Craveiro V., Guller S. and Mor G. (2013) Effect of culture conditions on the phenotype of THP-1 monocyte cell line. Am. J. Reprod. Immunol. 70, 80–86 10.1111/aji.12129 23621670 PMC3703650

[3] Basatemur G.L., Jorgensen H.F., Clarke M.C.H., Bennett M.R. and Mallat Z. (2019) Vascular smooth muscle cells in atherosclerosis. Nat. Rev. Cardiol. 16, 727–744 10.1038/s41569-019-0227-9 31243391

[4] Bekkering S., Quintin J., Joosten L.A., van der Meer J.W., Netea M.G. and Riksen N.P. (2014) Oxidized low-density lipoprotein induces long-term proinflammatory cytokine production and foam cell formation via epigenetic reprogramming of monocytes. Arterioscler. Thromb. Vasc. Biol. 34, 1731–1738 10.1161/ATVBAHA.114.303887 24903093

[5] Bennett M.R., Sinha S. and Owens G.K. (2016) Vascular smooth muscle cells in atherosclerosis. Circ. Res. 118, 692–702 10.1161/CIRCRESAHA.115.306361 26892967 PMC4762053

[6] Bentzon J.F., Otsuka F., Virmani R. and Falk E. (2014) Mechanisms of plaque formation and rupture. Circ. Res. 114, 1852–1866 10.1161/CIRCRESAHA.114.302721 24902970

[7] Bobryshev Y.V. (2006) Monocyte recruitment and foam cell formation in atherosclerosis. Micron 37, 208–222 10.1016/j.micron.2005.10.007 16360317

[8] Camnitz W., Burdick M.D., Strieter R.M., Mehrad B. and Keeley E.C. (2012) Dose-dependent effect of statin therapy on circulating CXCL12 levels in patients with hyperlipidemia. Clin. Transl. Med. 1, 23 10.1186/2001-1326-1-23 23369699 PMC3560987

[9] Chanput W., Mes J.J. and Wichers H.J. (2014) THP-1 cell line: an *in vitro* cell model for immune modulation approach. Int. Immunopharmacol. 23, 37–45 10.1016/j.intimp.2014.08.002 25130606

[10] Chatterjee M., Borst O., Walker B., Fotinos A., Vogel S., Seizer P. et al. (2014) Macrophage migration inhibitory factor limits activation-induced apoptosis of platelets via CXCR7-dependent Akt signaling. Circ. Res. 115, 939–949 10.1161/CIRCRESAHA.115.305171 25266363

[11] Chatterjee M., von Ungern-Sternberg S.N., Seizer P., Schlegel F., Buttcher M., Sindhu N.A. et al. (2015) Platelet-derived CXCL12 regulates monocyte function, survival, differentiation into macrophages and foam cells through differential involvement of CXCR4-CXCR7. Cell Death Dis. 6, e1989 26583329 10.1038/cddis.2015.233 PMC4670914

[12] Chavez-Sanchez L., Garza-Reyes M.G., Espinosa-Luna J.E., Chavez-Rueda K., Legorreta-Haquet M.V. and Blanco-Favela F. (2014) The role of TLR2, TLR4 and CD36 in macrophage activation and foam cell formation in response to oxLDL in humans. Hum. Immunol. 75, 322–329 10.1016/j.humimm.2014.01.012 24486576

[13] Chistiakov D.A., Melnichenko A.A., Myasoedova V.A., Grechko A.V. and Orekhov A.N. (2017) Mechanisms of foam cell formation in atherosclerosis. J. Mol. Med. 95, 1153–1165 10.1007/s00109-017-1575-8 28785870

[14] Daigneault M., Preston J.A., Marriott H.M., Whyte M.K. and Dockrell D.H. (2010) The identification of markers of macrophage differentiation in PMA-stimulated THP-1 cells and monocyte-derived macrophages. PLoS ONE 5, e8668 10.1371/journal.pone.0008668 20084270 PMC2800192

[15] Derakhshan R., Arababadi M.K., Ahmadi Z., Karimabad M.N., Salehabadi V.A., Abedinzadeh M. et al. (2012) Increased circulating levels of SDF-1 (CXCL12) in type 2 diabetic patients are correlated to disease state but are unrelated to polymorphism of the SDF-1beta gene in the Iranian population. Inflammation 35, 900–904 10.1007/s10753-011-9391-8 21968974

[16] Doring Y., Noels H., van der Vorst E.P.C., Neideck C., Egea V., Drechsler M. et al. (2017) Vascular CXCR4 limits atherosclerosis by maintaining arterial integrity: evidence from mouse and human studies. Circulation 136, 388–403 10.1161/CIRCULATIONAHA.117.027646 28450349 PMC5777319

[17] Fong G.H. (2015) Potential contributions of intimal and plaque hypoxia to atherosclerosis. Curr. Atheroscler. Rep. 17, 510 10.1007/s11883-015-0510-0 25876920

[18] Gao J.H., Yu X.H. and Tang C.K. (2019) CXC chemokine ligand 12 (CXCL12) in atherosclerosis: an underlying therapeutic target. Clin. Chim. Acta 495, 538–544 10.1016/j.cca.2019.05.022 31145896

[19] Jie W., Wang X., Zhang Y., Guo J., Kuang D., Zhu P. et al. (2010) SDF-1alpha/CXCR4 axis is involved in glucose-potentiated proliferation and chemotaxis in rat vascular smooth muscle cells. Int. J. Exp. Pathol. 91, 436–444 10.1111/j.1365-2613.2010.00720.x 20586815 PMC3003841

[20] Kattoor A.J., Goel A. and Mehta J.L. (2019) LOX-1: regulation, signaling and its role in atherosclerosis. Antioxidants (Basel) 8, 10.3390/antiox8070218 31336709 PMC6680802

[21] Kumar R., Tripathi V., Ahmad M., Nath N., Mir R.A., Chauhan S.S. et al. (2012) CXCR7 mediated Gialpha independent activation of ERK and Akt promotes cell survival and chemotaxis in T cells. Cell. Immunol. 272, 230–241 10.1016/j.cellimm.2011.09.015 22070874

[22] Lacolley P., Regnault V., Nicoletti A., Li Z. and Michel J.B. (2012) The vascular smooth muscle cell in arterial pathology: a cell that can take on multiple roles. Cardiovasc. Res. 95, 194–204 10.1093/cvr/cvs135 22467316

[23] Li L.X., Zhang X.F., Bai X. and Tong Q. (2013) SDF-1 promotes ox-LDL induced vascular smooth muscle cell proliferation. Cell Biol. Int. 37, 988–994 10.1002/cbin.10126 23658061

[24] Li X., Zhu M., Penfold M.E., Koenen R.R., Thiemann A., Heyll K. et al. (2014) Activation of CXCR7 limits atherosclerosis and improves hyperlipidemia by increasing cholesterol uptake in adipose tissue. Circulation 129, 1244–1253 10.1161/CIRCULATIONAHA.113.006840 24374972

[25] Libby P., Bornfeldt K.E. and Tall A.R. (2016) Atherosclerosis: successes, surprises, and future challenges. Circ. Res. 118, 531–534 10.1161/CIRCRESAHA.116.308334 26892955 PMC4762065

[26] Merched A.J., Ko K., Gotlinger K.H., Serhan C.N. and Chan L. (2008) Atherosclerosis: evidence for impairment of resolution of vascular inflammation governed by specific lipid mediators. FASEB J. 22, 3595–3606 10.1096/fj.08-112201 18559988 PMC2537438

[27] Merckelbach S., van der Vorst E.P.C., Kallmayer M., Rischpler C., Burgkart R., Doring Y. et al. (2018) Expression and cellular localization of CXCR4 and CXCL12 in human carotid atherosclerotic plaques. Thromb. Haemost. 118, 195–206 10.1160/TH17-04-0271 29304539

[28] Moore K.J., Sheedy F.J. and Fisher E.A. (2013) Macrophages in atherosclerosis: a dynamic balance. Nat. Rev. Immunol. 13, 709–721 10.1038/nri3520 23995626 PMC4357520

[29] Olive M., Mellad J.A., Beltran L.E., Ma M., Cimato T., Noguchi A.C. et al. (2008) p21Cip1 modulates arterial wound repair through the stromal cell-derived factor-1/CXCR4 axis in mice. J. Clin. Invest. 118, 2050–2061 18464929 10.1172/JCI31244 PMC2373418

[30] Qin Z. (2012) The use of THP-1 cells as a model for mimicking the function and regulation of monocytes and macrophages in the vasculature. Atherosclerosis 221, 2–11 10.1016/j.atherosclerosis.2011.09.003 21978918

[31] Rafieian-Kopaei M., Setorki M., Doudi M., Baradaran A. and Nasri H. (2014) Atherosclerosis: process, indicators, risk factors and new hopes. Int. J. Prev. Med. 5, 927–946 25489440 PMC4258672

[32] Rensen S.S., Doevendans P.A. and van Eys G.J. (2007) Regulation and characteristics of vascular smooth muscle cell phenotypic diversity. Netherlands Heart J. 15, 100–108 10.1007/BF03085963 17612668 PMC1847757

[33] Schiraldi M., Raucci A., Munoz L.M., Livoti E., Celona B., Venereau E. et al. (2012) HMGB1 promotes recruitment of inflammatory cells to damaged tissues by forming a complex with CXCL12 and signaling via CXCR4. J. Exp. Med. 209, 551–563 10.1084/jem.20111739 22370717 PMC3302219

[34] Schober A., Hristov M., Kofler S., Forbrig R., Lohr B., Heussen N. et al. (2011) CD34+CD140b+ cells and circulating CXCL12 correlate with the angiographically assessed severity of cardiac allograft vasculopathy. Eur. Heart J. 32, 476–484 10.1093/eurheartj/ehq402 21036775

[35] Sjaarda J., Gerstein H., Chong M., Yusuf S., Meyre D., Anand S.S. et al. (2018) Blood CSF1 and CXCL12 as causal mediators of coronary artery disease. J. Am. Coll. Cardiol. 72, 300–310 10.1016/j.jacc.2018.04.067 30012324

[36] Tavakolian Ferdousie V., Mohammadi M., Hassanshahi G., Khorramdelazad H., Khanamani Falahati-Pour S., Mirzaei M. et al. (2017) Serum CXCL10 and CXCL12 chemokine levels are associated with the severity of coronary artery disease and coronary artery occlusion. Int. J. Cardiol. 233, 23–28 10.1016/j.ijcard.2017.02.011 28189264

[37] Wang J.C. and Bennett M. (2012) Aging and atherosclerosis: mechanisms, functional consequences, and potential therapeutics for cellular senescence. Circ. Res. 111, 245–259 10.1161/CIRCRESAHA.111.261388 22773427

[38] Yin Y.W., Liao S.Q., Zhang M.J., Liu Y., Li B.H., Zhou Y. et al. (2015) TLR4-mediated inflammation promotes foam cell formation of vascular smooth muscle cell by upregulating ACAT1 expression. Cell Death Dis. 6, 1659 25719242 10.1038/cddis.2015.35 PMC4669797

[39] Zhang M., Qiu L., Zhang Y., Xu D., Zheng J.C. and Jiang L. (2017) CXCL12 enhances angiogenesis through CXCR7 activation in human umbilical vein endothelial cells. Sci. Rep. 7, 8289 10.1038/s41598-017-08840-y 28811579 PMC5557870

[40] Zhou B.R., Pan Y. and Zhai Z.M. (2011) Fibrinogen and P-selectin expression in atherosclerosis model of Sprague Dawley rat. Chin. Med. J. 124, 3768–3772 22340239

