# Correction: Foam cells promote atherosclerosis progression by releasing CXCL12

**DOI:** 10.1042/BSR-2019-3267_COR

**Published:** 2022-05-04

**Keywords:** Atherosclerosis, CXCL12, foam cell, macrophage

The authors of the original article “Foam cells promote atherosclerosis progression by releasing CXCL12” (*Biosci Rep* (2020) **40**(1):BSR20193267; https://doi:10.1042/BSR20193267) would like to correct an error in Figure 3C. The authors found that the third image of second row in Figure 3C is overlaps with the of the first image in the fourth row of Figure 4B. This error had been introduced during figure preparation for their manuscript's submission to the journal. The correct version of this image is present in this Correction. The authors declare that this Correction does not alter the conclusions of the study.

**Figure 3:**
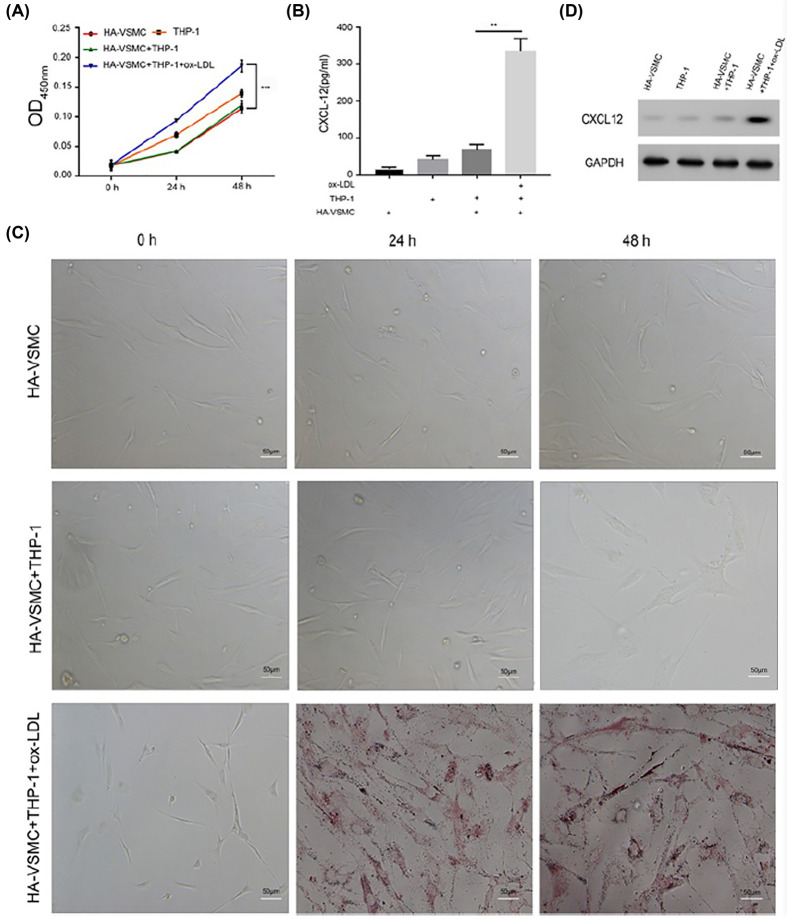
Ox-LDL-treated THP-1 cells promoted HA-VSMC proliferation and foam cell formation (**A**) HA-VSMCs, THP-1 cells, co-cultured HA-VSMCs (bottom chamber) with THP-1 cells (upper chamber), and co-cultured HA-VSMCs (bottom chamber) with ox-LDL-treated THP-1 cells (upper chamber) were seeded into Transwell plates and incubated for 24 or 48 h. The upper chamber was then removed and cell proliferation was detected with the MTT assay. (**B**) Cells were treated as in (A); after 48 h, the upper chamber was removed and the HA-VSMCs were fixed and stained with Oil Red O. The respective images are shown (×200). (**C**) Cells were treated as in (A); after 48 h, the upper chamber was removed, the culture medium in the bottom chamber was collected, and CXCL12 expression was examined by ELISA. (**D**) Cells were collected and lysed with lysis buffer, and Western blotting was performed. **, *P<*0.05, HA-VSMC+THP-1 *s.* HA-VSMC+THP-1+ox-LDL; ***, *P*<0.001, HA-VSMCs+THP-1 *vs.* HA-VSMCs+THP-1+ox-LDLs.

